# Nodular fasciitis: a comprehensive, time-correlated investigation of 17 cases

**DOI:** 10.1038/s41379-021-00883-x

**Published:** 2021-08-11

**Authors:** Zoltán Sápi, Zoltán Lippai, Gergő Papp, Lajos Hegyi, Johanna Sápi, Katalin Dezső, Károly Szuhai

**Affiliations:** 1 1st Department of Pathology and Experimental Cancer Research, Semmelweis Univesity, Budapest, Hungary; 2 Research and Innovation Center of Óbuda University, Physiological Controls Group, Óbuda University, Budapest, Hungary; 3 Department of Cell and Chemical Biology, Leiden University Medical Center, Leiden, the Netherlands; 4 HCEMM-SE Molecular Oncohematology Research Group, 1st Department of Pathology and Experimental Cancer Research, Semmelweis University, Budapest, Hungary

**Keywords:** Oncogenesis, Translational research

## Abstract

The self-limited nature of nodular fasciitis (NF) is well-known but its precise mechanism has not yet been clarified. We observed that “young” NF (preoperative duration <1 month) consistently contains a higher percentage (~80%) of USP6 break-apart FISH signals than “old” NF (preoperative duration >3 months) (~20%). Thus, we hypothesized that our original observation may reflect a connection with the self-limited nature of NF. Seventeen cases with reliable data concerning the onset were selected, thus approximating the lifetime of each tumor. Besides the *USP6* interphase FISH examination, we also checked the most common *MYH9-USP6* fusion using RT-PCR. Because of the known pathways of the tumorigenesis of NF, the mRNA level of *USP6*, *TRAIL*, *IFN-beta*, *JAK1*, *STAT1*, *STAT3*, *JUN*, and *CDKN2A* was measured using qRT-PCR. Regarding proteins, USP6, p16, p27, TRAIL, and IFN-beta were examined using immunohistochemistry. Targeted gene panel next-generation sequencing (NGS) of three cases was additionally performed. We found a strong negative correlation (*p* = 0.000) between the lifetime and percentage of USP6 break-apart signals and a strong positive relationship (*p* = 0.000) between USP6 break-apart signals and mitotic counts. Results of immunostainings, along with qRT-PCR results, favored the previously-suggested USP6-induced negative feedback mechanism through activation of TRAIL and IFN-beta, likely resulting in apoptosis and senescence of tumor cells harboring *USP6* fusions. Targeted-NGS resulted in the detection of several variants, but no additional recurrent changes in the pathogenesis of these tumors. We revealed on a cellular level the USP6-induced negative feedback mechanism. In conclusion, we emphasize that in “old” NF, the percentage of *USP6* break-apart FISH signals can be as low as 14–27% which can be very important from a differential diagnostic point of view. We emphasize that a careful examination and interpretation of the NGS data is needed before clinical decision-making on treatment.

## Introduction

NF is a typically rapidly growing but benign self-limited myofibroblastic/fibroblastic tumor^1^. The most common localization is the surface of the fascia of upper extremities, trunk, and head and neck, but rarely intra-articular, intravascular, dermal involvement has been described^2–4^. Preoperative duration in most cases is less than 3 months, but “old” lesions are not so rare and they have sometimes prominent fibrosis and hyalinized areas^1,5^. Because these “old” cases can cause differential diagnostic problems, we regularly performed *USP6* FISH as a desirable diagnostic criterion—as recommended by the latest WHO “Soft Tissue and Bone Tumors” book^1^—with our original observation that the percentage of *USP6* break-apart signals of “old” NF were much lower compared with “young” NF. This observation prompted us to investigate whether it might have shown a connection with a self-limited nature. Concerning the pathogenesis of NF, it is well known that USP6 activation by promoter-swapping (usually *MYH9* being the partner gene) is characteristic with involvement of JAK-1/STAT1/STAT3 pathways resulting in stabilization of the JUN protein^6–12^. Recently, it was reported that cellular senescence through CDKN2A/p16 could be responsible for self-limited nature, and even more, interestingly, a negative feedback mechanism, where USP6 induces TRAIL-mediated apoptosis, was discovered^13,14^. We aimed to investigate the above-mentioned mechanisms on a cellular level and for this purpose, we selected 17 cases of NF with reliable data concerning the onset; thus, approximating the lifetime of each tumor. Because it is very rare to gain insight into possible unknown gene alterations in a benign tumor, we selected three cases for this purpose (using NGS) as to whether we could find any alteration and whether there is any difference between “young” and “old” NF.

## Materials and methods

### Case selection

Seventeen cases of NF from our institutional and consultation archives (diagnosed between 2015 and 2020) were involved in this study. Reliable data about the onset of each case was available. If the lifetime of NF was less than one month, we designated it as “young” and if it was more than 3 months, we designated it as “old” NF. Cases between 1 and 3 months were termed “in-between”. In 6 cases (mainly of “old” NF), aspiration cytology was performed; in each case with a suggested diagnosis of NF. Because both the date of aspiration and the surgical removal was known (mean time from aspiration to removal was 2.3 months), it helped to select those NF cases which were over 3 months concerning their lifetime. The diagnosis was based on typical clinical features as well as characteristic morphology. All cases showed strong alpha-smooth muscle actin positivity while H-Caldesmon, Desmin, CD34, and S100 were negative. The site, age, and gender distribution (9–52 years, 11 male, 6 females) is shown in Table 1. As a control for the gene expression comparisons at mRNA level with qRT-PCR, a case of deep fibrous histiocytoma as a control (22-year-old female; a deep subcutaneous tumor on left shoulder; size was 2 × 1.5 cm; it was CD34 and alpha-smooth muscle actin positive but negative with STAT6, Desmin, H-Caldesmon and ALK1; no split-signal of *USP6* break-apart probe was recognized) was selected. Two bone and soft tissue pathologists (ZS and KD) reviewed all cases. Main clinical information was obtained from the medical record register of our institution and is summarized in Table 1. Our institutional review board approved this study (TUKEB 155/2012).

**Table 1:** Summary of clinical data and PCR results of the 17 patients with nodular fasciitis.

Case number	Age (years), sex	Site	FISH USP6 (%)	Time^a^ (months)	Mitosis/10 HPF	Previous aspiration cytology	Size at the time of aspiration cytology (mm)	Size of the surgical specimen (mm)	USP6	TRAIL	IFNB	JAK1	STAT1	STAT3	JUN	CDKN2A	MYH9-USP6 fusion
1	52/M	l. thigh	14	>3	0.5	Yes	45	21	2548	3.8	3.3	2.0	5.7	1.2	1.6	3.8	no
2	40/M	r. little finger	17	>3	0.5	Yes	20	9	2954	3.3	1.7	2.9	8.2	1.0	2.2	0.4	yes
3	28/F	face	18	2–3	1	Yes	20	16	1592	1.9	1.8	1.6	3.8	1.0	1.9	2.9	yes
4	44/M	l. arm	20	>3	1	Yes	n.a.	10	12863	1.1	17.8	3.1	8.9	1.5	5.1	0.6	yes
5	9/M	chest	27	>3	1	No	–	13	5169	5.0	6.7	2.4	10.8	1.7	3.8	5.8	yes
6	52/M	l. knee	33	2–3	1	Yes	27	20	2099	2.5	7.1	3.6	10.1	1.8	6.7	0.3	no
7	21/F	forehead	38	2–3	2	No	–	5	2533	2.0	4.0	1.7	5.3	1.2	0.8	1.2	no
8	5/M	r. gluteal region	46	2–3	5	No	–	12	2681	6.4	1.2	1.5	6.0	0.9	1.1	1.6	yes
9	42/F	l. shoulder blade	47	2–3	3	No	–	9	82	2.2	1.0	2.4	3.6	1.5	4.2	3.6	yes
10	42/F	forehead	52	1–2	3	No	–	8	398	3.1	1.0	1.8	2.4	1.1	0.6	1.7	no
11	17/F	neck	58	1–2	6	No	–	7	144	2.8	7.8	1.2	11.0	1.1	1.5	2.1	yes
12	21/M	r. shoulder	61	2–3	4	Yes	34	25	1206	2.9	0.8	1.7	5.0	1.3	1.4	1.9	yes
13	6/M	r. supraclav. region	63	1–2	3	No	–	18	1303	3.9	0.8	1.6	4.4	1.5	0.6	1.0	yes
14	12/M	face	78	<1	5	No	–	25	1865	2.8	1.2	1.4	6.8	0.7	7.3	2.2	yes
15	25/M	l. forearm	80	<1	10	No	–	15	159	1.1	14.1	1.5	1.4	0.6	1.6	5.7	no
16	40/F	r. forearm	84	<1	11	No	–	17	2317	0.6	6.4	2.0	1.4	1.0	2.6	3.0	no
17	33/M	r. forearm	91	<1	18	No	–	15	165	0.7	4.2	1.4	1.1	1.0	1.3	1.2	yes
									2358	2.7	4.8	2.0	5.7	1.2	2.6	2.3	

*NA* not available.

^a^Time elapsed between the onset and surgical removal.

### Immunohistochemistry

Immunohistochemistry was performed on deparaffinized, rehydrated sections obtained from a representative formalin-fixed, paraffin-embedded block from each case using antibody-specific epitope retrieval techniques with the Bond-Max (Leica Biosystems, Wetzlar, Germany) automated system for the detection of the following antigens: p27 (Santa Cruz, rabbit polyclonal IgG, 1:50–1:500), p16 (Roche, clone E6H4, ready to use), USP6 (Abcam, rabbit polyclonal IgG, 1:1000), IFNB (Abcam, rabbit polyclonal IgG, 1:100), TRAIL (Abcam, rabbit polyclonal IgG, 1:1000). The immunohistochemical results were scored both for staining intensity and cellular localization (membranous, cytoplasmic, nuclear) of the different proteins and were further correlated for differences between “young” and “old” NF (Supplementary Table 1).

### Fluorescence in situ hybridization (FISH)

The FISH analysis was performed on interphase nuclei of paraffin-embedded 4μm-sections using ZytoLight SPEC dual-color break-apart probe specific for *USP6* at 17p13.2 with Spectrum green™ telomeric (5′ to the breakpoint) and Spectrum orange™ centromeric (3′ to the breakpoint) labeling (ZytoVision, Bremerhaven, Germany). In each case, at least 100 tumor cells were counted (excluding obvious endothelial and inflammatory cells) and the percentage of cells harboring split green and red signals was given. FISH signals were scored by one pathologist and one biologist (ZS and GP). The individual cases were arranged in ascendant order based on the percentage of split signals.

### mRNA isolation and quantitative reverse transcriptase-polymerase chain reaction (qRT-PCR)

Total RNA was extracted from formalin-fixed, paraffin-embedded scrolls using the RNeasy FFPE Kit (Qiagen, Hilden, Germany), as per the manufacturer’s instructions. Reverse transcription was performed using the High-Capacity cDNA Reverse Transcription Kit (Thermo Fisher Scientific, Waltham, MA, USA), as per the manufacturer’s instructions. For the qRT-PCR, we used the following TaqMan Gene Expression Assays (Thermo Fisher Scientific): *USP6*, *JAK1*, *STAT1*, *STAT3*, *IFNB*, *JUN*, *CDKN2A*, and *GUSB* as well as *HPRT1* as control genes. Cycling conditions were 50 °C for 10 min, then 95 °C for 10 min, followed by 45 cycles of 95 °C for 15 s, 60 °C for 60 s. All samples were normalized using the delta-delta CT method against a case of deep fibrous histiocytoma. Normalization of real-time PCR was performed as previously described by Vandesompele et al.^15^ (Supplementary Table 2). For the detection of *MYH9*(exon1)-*USP6*(exon1) and *MYH9*(exon1)-*USP6*(exon2) fusions, on-demand Custom Plus TaqMan RNA Assays (Thermo Fisher Scientific) were used.

### Next-generation sequencing

Three cases (Nr1, 14, and 15, representing both “old” and “young” fasciitis) were chosen for DNA and RNA-based sequencing with TruSight™ Oncology and TruSight Oncology 500 High-Throughput (Illumina, San Diego, CA, USA). DNA was extracted from formalin-fixed, paraffin-embedded scrolls using the QIAamp DNA FFPE Tissue Kit (Qiagen), as per the manufacturer’s instructions. Total RNA was extracted from formalin-fixed, paraffin-embedded scrolls using the RNeasy FFPE Kit (Qiagen), as per the manufacturer’s instructions. The comprehensive genomic profiling included the analysis of 523 genes for insertion, deletion, single nucleotide variant, copy number variation, and 55 genes for known and novel fusions and splice variants. The panel also enabled the detection of microsatellite instability and tumor mutational burden. The Genome Reference Consortium Human Build 37 (hg19) reference version of the genome was used for the analysis. Reports were generated using Clinical Insight (QCI^TM^; Qiagen) variant analysis, interpretation, and decision support tool for research and clinical labs analyzing human genetics data. QCI Interpret software includes the following underlying databases, data reference sets, and tools; QIAGEN Clinical Insight-Interpret (7.1.20210316), Ingenuity Knowledge Base (B-release), CADD (v1.6), Allele Frequency Community (2019-09-25), EVS (ESP6500SI-V2), Refseq Gene Model (2020-04-06), JASPAR (2013-11), Ingenuity Knowledge Base Snapshot Timestamp (2021-03-18 14:15:50.073), Vista Enhancer hg18 (2012-07), Vista Enhancer hg19 (2012-07), Clinical Trials (B-release), MITOMAP: A Human Mitochondrial Genome Database. http://www.mitomap.org, 2019 (2020-06-19), PolyPhen-2 (v2. 2.2), 1000 Genome Frequency (phase3v5b), ExAC (0.3.1), iva (Nov 20 02:39), TargetScan (7.2), phyloP hg18 (NCBI36 (hg18) 2009-11, GRCh37 (hg19) 2014-02, GRCh38 2015-05), phyloP hg19 (NCBI36 (hg18) 2009-11, GRCh37 (hg19) 2014-02, GRCh38 2015-05), GENCODE (Release 33), CentoMD (5.3), OMIM (July 06, 2020), gnomAD (2.1.1), BSIFT (2016-02-23), TCGA (2013- 09-05), Clinvar (2020-09-15), DGV (2016-05-15), COSMIC (v92), HGMD (2020.4), OncoTree (oncotree_2019_03_01), dbSNP (NCBI36 (hg18) 151, GRCh37 (hg19) 153, GRCh38 153), SIFT4G (2016-02-23).

### Statistical analysis

To investigate the relationship between *USP6*-FISH split signal (%) and NF properties: the lifetime of NF, mitosis, and size, as well as different gene expressions (*USP6*, *TRAIL*, *IFNB*, *JAK1*, *STAT1*, *STAT3*, *JUN*, and *CDKN2A*), statistical analyzes were carried. All calculations were made comparing the USP6 FISH percentage with other parameters using simple linear regression. Matlab R2020b (MathWorks, USA) was used to calculate correlation and p values, and to visualize the results. The correlation was investigated with Pearson’s rank correlation test (Pearson’s R) and coefficient of determination (*R*^2^), linear regression models were plotted with 95% confidence bounds. The estimated lifetime of NF was statistically modified to be able to carry out statistical analysis (Supplementary Table 3).

## Results

### Morpho-molecular characteristics of the tumors

**Fig. 1:**
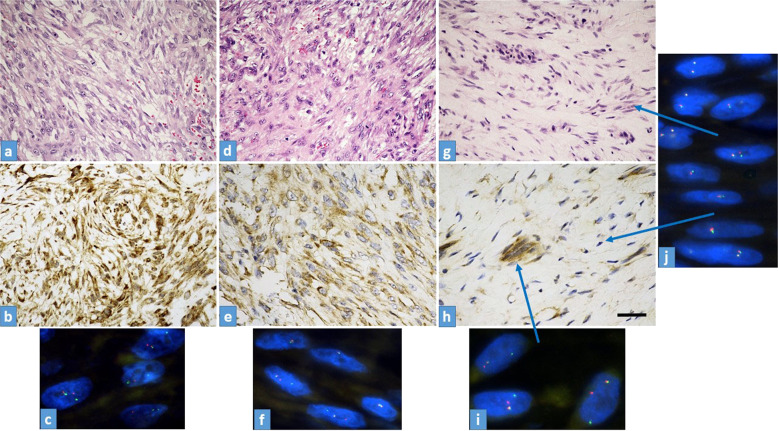
Morpho-molecular characteristics of the tumors. **a**–**c** Case No15; “young” NF (*USP6*-FISH: 80%). **a** H&E stain shows a highly cellular tumor with spindle or stellate cells arranged in a loose fascicular pattern. **b** Strong, diffuse, and granular cytoplasmic USP6 immunostaining (**b**) along with evenly distributed FISH break-apart signals containing tumor cells. **c** USP6-FISH reaction, all cells in the picture contain split signals. **d**–**f** Case No9; “in between” NF (USP6-FISH: 47%). **d** H&E stain shows plump but regular spindle-shaped fibroblasts or myofibroblasts. **e** A mixed population of USP6 positive and negative tumor cells (USP6 immunostaining). **f** USP6-FISH-reaction shows two tumor cells with split signals and three with no split signals. **g**–**j** Case No1; “old” NF (USP6-FISH: 14%). **g** H&E stain shows a tumor with low cellularity, composed of plump and spindle cells in a hyalinized fibrous stroma. **h** USP6 immunohistochemistry demonstrates a strong, but focal cytoplasmic positivity, with positive cells usually arranged in smaller groups **i**. FISH-USP6 split signals localized in small groups of USP6 immunopositive tumor cells (arrow). **j** Tumor cells with no FISH-USP6 split signals are distributed evenly in the hyalinized background (arrows). Scale bar for Fig. 1—50 µm.

“Young” tumors are hypercellular and they are mostly composed of haphazardly arranged mass of plump cells with a tissue-culture-like growth pattern. The cells are set in a fibromyxoid stroma. “In between” fasciitis is characterized by the presence of spindle and plump tumor cells. “Old” tumors are characterized by a collagen-rich stroma. The majority of the tumor cells are spindle-shaped but small groups of plump tumor cells are also visible (Fig. 1).

### Fluorescence in situ hybridization (USP6 FISH)

**Fig. 2:**
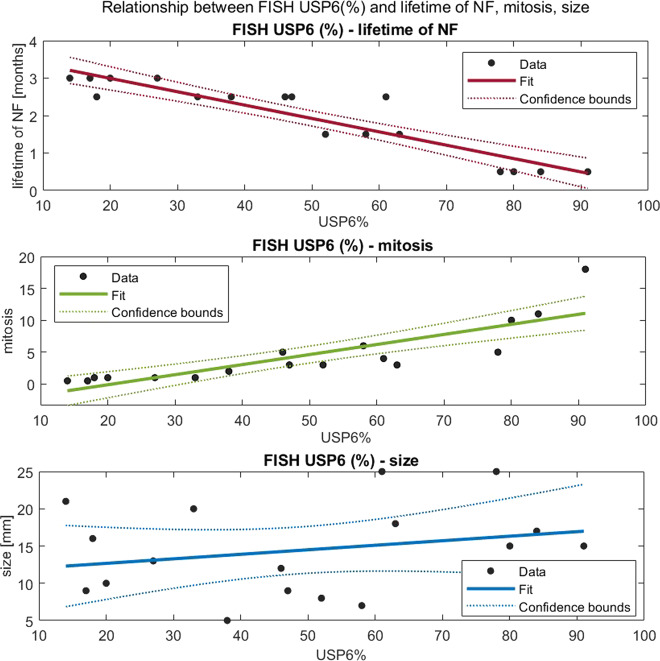
Relationship between USP6-FISH (%) and lifetime of NF, mitosis, size. **a** Linear regression between USP6-FISH (%) and lifetime of NF: *R* = −0.921 (strong negative relationship), *R*^2^ = 0.849, *p* = 0.000 (significant with significance level 0.05). **b** Linear regression between USP6-FISH (%) and mitosis: *R* = 0.848 (strong positive relationship), *R*^2^ = 0.719, *p* = 0.000 (significant with significance level 0.05). **c** Linear regression between USP6-FISH (%) and size: *R* = 0.251 (no relationship), *R*^2^ = 0.063, *p* = 0.330 (not significant with significance level).

The percentage distribution of the split signal ranged from 14% to 91% (Table 1). In “young” NF, this range was between 78% and 91% and in “old” NF, this was between 14 and 27%. A strong negative relationship (*R* = −0.921) was found between the split-signal percentage and appearance (Fig. 2) which means the “younger” the NF is, the higher the percentage. This also implies that with time, there are fewer proliferating cells which were proved by the strong positive relationship (*R* = 0.848) between the split-signal percentage and mitotic activity (Fig. 2). In “young” NF the cells with split-signals were evenly distributed (Fig. 1c), while in “old” fasciitis, this distribution was focal. Cells in the more hyalinized area were usually negative indicating the presence of non-neoplastic stromal cells, while small groups of plump neoplastic cells were positive (Fig. 1i–j). No relationship (*R* = 0.238) between the split-signal percentage and tumor size was observed (Fig. 2).

### Real-time polymerase chain reaction

**Fig. 3:**
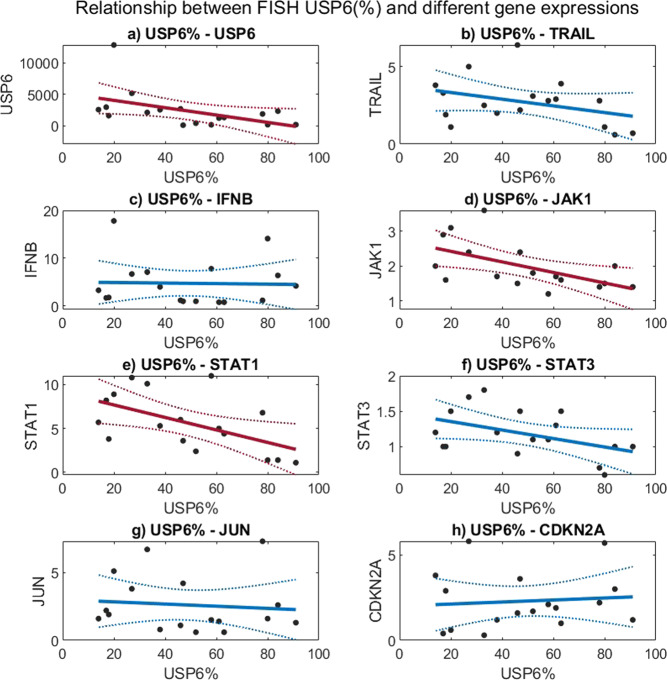
Relationship between USP6-FISH (%) and different gene expressions (using relative gene expression). Dots represent data points, solid line is the fitted curve, and dashed lines are confidence bounds. The significance level is 0.05, significant regressions (with negative relationship) are marked with red lines. **a** Linear regression between USP6-FISH(%) and *USP6*: R = −0.488, *p* = 0.047. **b** Linear regression between USP6-FISH(%) and *TRAIL*: *R* = −0.357, *p* = 0.160. **c** Linear regression between USP6-FISH(%) and *IFNB*: *R* = −0.029, *p* = 0.911. **d** Linear regression between USP6-FISH(%) and *JAK1*: *R* = −0.566, *p* = 0.018. **e** Linear regression between USP6-FISH(%) and *STAT1*: *R* = −0.550, *p* = 0.022. **f** Linear regression between USP6-FISH(%) and *STAT3*: *R* = −0.454, *p* = 0.067. **g** Linear regression between USP6-FISH(%) and *JUN*: *R* = −0.097, *p* = 0.711. **h** Linear regression between USP6-FISH(%) and *CDKN2A*: *R* = 0.089, *p* = 0.734.

Only *USP6* mRNA showed tremendous overexpression (on average: 2358 times) while concerning the others (*TRAIL*, *IFNB*, *JAK1*, *STAT1*, *JUN*, and *CDKN2A*), only moderate overexpression was observed (average range: 2–5.7). *STAT3* was practically not overexpressed (on average: 1.2 times) (Table 1). Regarding the relationship between *USP6* split-signal percentage and different gene expressions, the percentage of cells with USP6 translocation showed a significant negative correlation with relative mRNA expression of *USP6*, *JAK1*, and *STAT1*; *p* values were *p* = 0.047, *p* = 0.018, and *p* = 0.022, respectively (Fig. 3). *P* values were not adjusted for multiple comparisons due to the fact that the comparisons are not independent (*p*-value adjustment methods can not be used in this case).

While *TRAIL* (*p* = 0.16) and *STAT3* (*p* = 0.06) showed a not significant negative correlation (Fig. 3), but *IFNB*, *JUN*, and *CDKN2A* displayed no correlation with USP6 FISH percentage at all; *p* values were *p* = 0.911, *p* = 0.711 and *p* = 0.734, respectively (Fig. 3). The relative gene expression values are shown in Table 1 and Supplementary Table 2. *MYH9-USP6* fusion was observed in 11 cases (64.7%) involving evenly “young” and “old” cases and “in-between” cases (Table 1).

### Immunohistochemistry

The localization, intensity, and expression percentage of immunohistochemical reactions are summarized in Supplementary Table 1.

*USP6*: a strong granular intracytoplasmic reaction was characteristic in each case. However, the distribution showed marked differences concerning “young”, “old” and in-between cases. In “young” NF (case numbers: 14–17), there was a diffuse positivity (Fig. 1b) while in “old” NF (case numbers: 1–2 and 4–5), we found only focal positivity; mainly localized on smaller groups with plump tumor cells. Elongated cells residing in hyalinized areas were usually negative (Fig. 1h). The immunohistochemistry confirmed that USP6 overexpression was characteristically confined to neoplastic cells harboring the USP6 split signals (Fig. 1h–i). Regarding the “in-between” cases (case numbers: 6–13 and 3), the distribution of positive and negative cells was found side by side; creating an evenly “mixed” population (Fig. 1e).

**Fig. 4:**
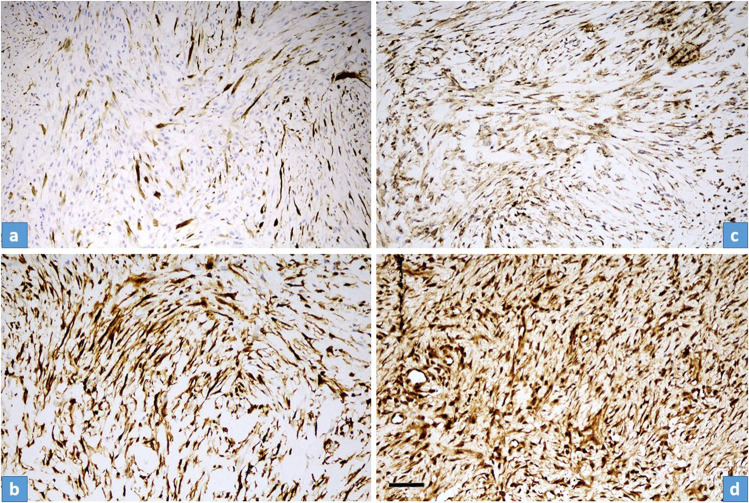
Distribution of p16 and p27 immunohistochemical stainings in “young” and “old” NF. **a**, **c** Strong but focal p16 ((**a**), case No14) and p27 ((**c**), case No16) cytoplasmic/nuclear reaction were characteristic for “young” fasciitis. **b**, **d** Diffuse strong p16 ((**b**), case No2) and p27 ((**d**), case No4) cytoplasmic/nuclear reaction were observed in “old” fasciitis. Scale bar for Fig. 4—100 µm.

*Senescence markers: p16 and p27*: a strong nuclear and cytoplasmic positivity was found in each case, but again there was a difference in the distribution. In “young” NF only focal positivity was observed which gradually turned into a diffuse positivity in “old” NF (Fig. 4).

**Fig. 5:**
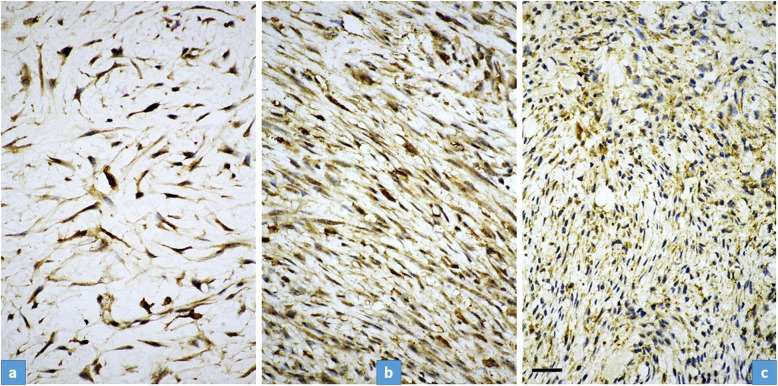
Immunostaining of TRAIL in “young” ((**a**), case No14), ‘in between’ ((**b**) case No10), and “old” NF ((**c**), case No2). Strong, mainly nuclear staining is characteristic for “young” fasciitis (**a**) while only membranous-cytoplasmic staining can be observed in “old” fasciitis (**c**). Concerning the “in between” cases, a mixed nuclear and membranous-cytoplasmic staining could be observed (**b**). The “young” fasciitis (same as in (**a**) and (**c**)) was much more cellular than the “old” (**c**) one, but we chose a loose, myxoid area for a better demonstration of nuclear staining. Scale bar for Fig. 5—50 µm.

*TRAIL*: both nuclear and membranous-cytoplasmic positivity was observed. The striking difference was, however, that in “young” NF, the positivity was characteristically nuclear and less intensively membranous-cytoplasmic while in “old” NF; it changed and there was practically only membranous-cytoplasmic positivity and no nuclear positivity (Fig. 5a–c). In-between cases displayed mixed nuclear and membranous-cytoplasmic positivity (Fig. 5b).

*IFNB*: unequivocal cytoplasmic positivity was found in each case. The only difference between “old” and “young” NF was that in the “old”, positivity was less intensive, especially in hyalinized areas (Supplementary Fig. 1).

### Next-generation sequencing (NGS)

Tumor samples were selected with a minimum of 50% tumor content based on visual estimation (50–80%). The automated reporting listed 58 variants in total (Supplementary Table 4) including 1 fusion event (28 for case 1, 12 for case 14, and 18 for case 15). Applying a filter that excludes samples with PolyPhen benign, gnomAD <5%, with less than ten read depth, and samples between 45% and 55% allele frequencies, eight variants remained. Distributions of these variants were: patient nr1: 4, nr14: 3, and nr15: 1. From these, promoters, UTR, and intronic variants were further filtered out. In all three cases, the reported variant allele frequency was low 1.5–4%.

Case 1: *PDGFRB* c.3251_3256delCAGAGC P1084_E1085del (The PDGFRB in-frame deletion has been described both in normal individuals) (last accessed 07-04-2021 https://gnomad.broadinstitute.org/variant/5-149495390-AGCTCTG-A?dataset=exac) and in various cancer samples with unknown significance^16^. The alteration is outside of the kinase domain and likely not affecting protein function.

Case 15: *FBXW7* c.1513C>T. p.R505C. The FBXW7 variant is a predicted likely pathogenic variant, reported in the COSMIC database under COSV55891274 Genomic Mutation ID. The FBXW7 mutation confers loss of interaction with its substrate resulting in loss of function. The found mutation leads to resistance in melanoma, colorectal cancer, and T cell leukemia using Pembroziluman, regorafenib, and MRK-003 treatments and a predicted sensitivity to vorinostat in a head and neck cancer cell line model (ref: https://ckb.jax.org/geneVariant/show?geneVariantId=916).

Case 14: *NOTCH2* c.2809T>A p.C937S so far this variant is unreported and likely to be damaging (Supplementary Table 4 red highlights).

In all three cases, the TMB (tumor mutation burden) was low and MSI was stable, with the highest value in case Nr:1 (2.4 mutations/megabase), in line with translocation-driven tumors. Detailed data are available in Supplementary Table 4.

## Discussion

After a fairly long debate, NF was considered as a true neoplasm and not as an inflammatory reactive lesion due to its consistent USP6 gene rearrangement; the most common is the MYH9-USP6 fusion^1,6,17–19^. Still, to its enigmatic nature belongs one side that NF is a self-limited tumor and on the other side that it looks like an aggressive neoplasm with rapid growth, an infiltrative growth pattern, and usually high mitotic rate^20^. In fact, the latter is underlined by the extremely rare event when the fusion gene is PPP6R3-USP6; resulting in a malignant transformation^21,22^, likely preventing NF from the usual spontaneous regression. However, in many instances before the spontaneous regression, the removal of NF happens due to the worrisome clinical picture, especially in children and young adults. Because this removal happens in different moments in the lifetime of nodular fasciitis, we collected 17 NF cases with reliable clinical data. Stratification was based on the time interval between onset and surgery as “young” (<1 month), “old” (>3 months), or “in-between” (1–3 months) (Table 1). In 6 cases, aspiration cytology was also performed which helped to undoubtedly determine the “old” cases (the suggestive diagnosis of NF using USP6-FISH on aspiration cytology resulted in a not urgent/delayed removal). Because “old” cases are sometimes also problematic from a differential diagnostic point of view (less classical picture with prominent hyalinization)^23^, we performed USP6-FISH. A “problematic” low percentage of USP6 split-signals (14% the lowest), in line with literature data with cut-off values as low as 20%^10^, was observed. This prompted us to systematically investigate the relationship between the proportion of USP6 split-signals and the age of NF. A strong relationship (*R* = −0.921) between the split-signal percentage and the lifetime of NF (Fig. 1) was observed which means the “younger” the NF is, the higher the percentage. The distribution of the proportion of split signal in “young” NF (78–91%) with an almost fully harmonic transition for the “in-between” cases to “old” NF (14–27%) was detected (Table 1). This gradual disappearance of the USP6 split-signals harboring neoplastic cells suggests that apoptosis and/or senescence might be involved in NF as the mechanism of the self-limited nature of nodular fasciitis. Similarly, marked differences in the distribution of cytoplasmic USP6 immunoreaction concerning “young”, “in-between”, and “old” cases were observed. In “young” NF a diffuse positivity prevailed (Fig. 3a) while in “old” NF, there was only focal positivity—mainly localized in smaller groups with plump tumor cells and no positivity in hyalinized areas with elongated tumor cells (Fig. 3c). Correlating the histology (morphology and mitotic activity) with the *USP6*-FISH pattern (*USP6* split-signals) along with USP6 immunohistology, the same distribution was observed. Immunohistochemistry confirmed that USP6 overexpression was characteristically confined to tumor cells harboring *USP6* split signals. Regarding the “in-between” cases, the distribution was even, where positive and negative cells were found side by side, creating a “mixed” population (Fig. 3b). Seemingly it is controversial that the mRNA level of *USP6* was increasing with time (*p* = 0.047), but presumably, in “old” NF, the fewer USP6 split-signal harboring tumor cells produced more mRNA as in the “young” ones. Gathering all this information together, it is evident that with time, the number of those tumor cells harboring *USP6* fusions are decreasing; suggesting some negative feedback mechanism triggered by USP6 fusion/overexpression. This theory is also supported by the fact that cases with aspiration cytology showed an unequivocal decrease in size (Table 1).

**Fig. 6:**
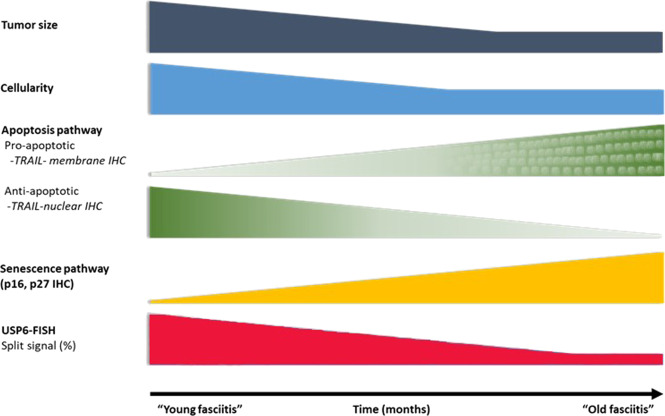
Schematic representation of the most important clinical-, morphological features and signaling pathways characterizing the “self-limited” nature of NF. Over time, NF displays a decrease in size (dark blue) and cellularity (pale blue). The number of the USP6 split signal harboring cells (red) and the expression of anti-apoptotic markers (TRAIL nuclear immunohistochemistry, green) also decrease, while the proapoptotic signals (TRAIL membrane immunohistochemistry, dotted-green) and senescence-related p27 and p16 expression (yellow) increase.

USP6-induced negative feedback mechanism through activation of *TRAIL* and *IFN*-*beta*, resulting in apoptosis, was suggested by Henrich et al.^14^, investigating Ewing sarcoma cell lines. They found that IFN-beta-induced TRAIL-mediated apoptosis was observed in USP6-positive but not USP6-negative Ewing sarcoma cells. In line with this, we observed an increased TRAIL expression both on the mRNA level and also a diffuse immunocytochemical reaction. However, very interestingly, in “young” NF the positivity was characteristically nuclear positivity and less intensively membranous-cytoplasmic, while in “old” NF, there was practically only membranous-cytoplasmic positivity and no nuclear positivity (Fig. 5). Nuclear localization of TRAIL can be regarded as hide-outs representing a strategy to resist TRAIL-mediated apoptosis^11^. In our “young” NF cases the nuclear localization of TRAIL can explain the early strong proliferative nature of NF, but the mechanism and how it is changing with time remains unknown. However, the dominant membranous-cytoplasmic TRAIL positivity in “old” NF may also explain that with time, the TRAIL-mediated apoptotic process starts. Expression of p16 in NF was described by Matsuda et al.^13^. They found variable, mainly a diffuse reaction, but a partial expression was also observed, although they did not stratify their cases by the lifetime of NF. In our cohort, in “young” NF, only focal positivity was observed and this focal positivity gradually passed through a diffuse positivity, most pronounced in “old” NF (Fig. 4). The same pattern was valid for p27 as another senescence marker. *CDKN2A* showed an increased but even expression level concerning the lifetime of NF (Fig. 2h). All this data speaks for the important role of senescence beginning very early and showing a full-blown picture at the end of regression of NF. Cellular senescence is a well-known tumor-suppressive mechanism that permanently arrests cells at risk for malignant transformation. Moreover, the senescence-associated secretory phenotype can regulate cell differentiation and stimulates cell motility. USP6 is a regulator of both cell migration and division^24^. The most common *MYH9-USP6* fusion was discovered by Erickson-Johnson et al.^6^ and they found a high expression level of *USP6* mRNA in their two examples of NF. We found *MYH9-USP6* fusion in 11 cases out of 17 (65%) which is very similar to others’ findings^6,10,25^ and the average mRNA expression of *USP6* (2358 times) was also very high. Interestingly, the average mRNA expression of *STAT1* was only moderately elevated (5.7 times) while *JAK1* (2 times) and *STAT1* (1.2 times) were not. These results confirm the role of the JAK-1/STAT1/STAT3 pathway and show that it is “active” during the whole lifetime of NF. Figure 6 summarizes the above discussed different mechanisms which may play a role in the self-limited nature of NF.

We sought to investigate any relationship between “young” and “old” NF and mutational status using targeted NGS. After standard reporting 58 variants were found in the three cases including one putative gene fusion (Supplementary Table 4). Using further filtering of the data, one putative somatic variant remained in each case. The standard reporting by commercial software tools resulted in 12 variants and actionable changes referring to clinical trials without a well-established link. This finding can be important because, nowadays, the concept of tumor-agnostic therapy became quite popular^26^. While the so-called tissue-agnostic therapy is well accepted, in our particular case of one NF the “recommended” Pembrolizumab treatment (POLE mutation, Supplementary Table 4— green highlight)^27^ would be obviously impractical. As expected, no recurrent mutation (including all 63 genes) of the cases examined (Supplementary Table 4) was found. This observation (numerous different not recurrent mutations) is in line with the notion that NF, as a “transient neoplasia”^1^, is somehow similar to mild/moderate epithelial dysplasia (with many different genetic alterations), still capable of regress^28^.

In conclusion, we emphasize that both “young” and “old” NF can cause differential diagnostic problems, the former because of sarcoma-like appearance, the latter because of the not-so-characteristic morphology. In both cases, one can confirm the diagnosis using USP6 FISH, but one has to be aware that in “old” NF, the percentage of USP break-apart FISH signals can be as low as 14–27%. We revealed on a cellular level likely the USP6-induced negative feedback mechanism resulting in regression. Overtesting and overinterpretation of NGS results may lead to unnecessary treatment burden and/or false hope for patients in NF misdiagnosed as malignant tumors.

## Supplementary information

Supplemental material

## Data Availability

The datasets used and/or analyzed during the current study are available from the corresponding author on reasonable request.
