# Supplementary material for: Nodular fasciitis: a comprehensive, time-correlated investigation of 17 cases
Source: Mod Pathol. 2021 Aug 11;34(12):2192–9. doi: 10.1038/s41379-021-00883-x (PMC8592838; doi:10.1038/s41379-021-00883-x)
Supplement: Supplementary file 1 — Supplemental material [file 41379_2021_883_MOESM1_ESM.pdf]

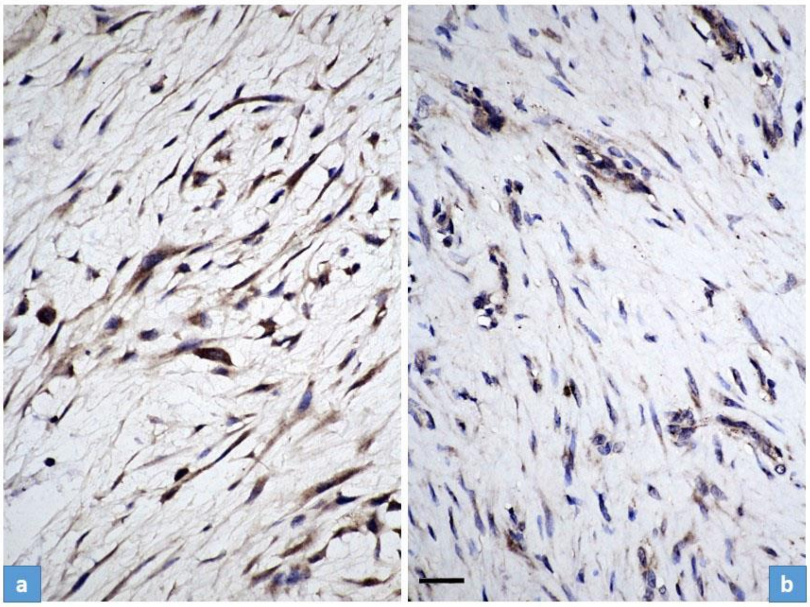

Supplementary Figure 1. Immunostaining of IFNB in ‘young’ (a, case No15) and in ’old’ NF (b, case No6). Unequivocal cytoplasmic positivity was found in both cases. Scale bar for Supplementary Figure 1. - 50µm

Supplementary Table 1 - Immunohistochemistry results

case number	USP6	p27	p16	IFNB +	IFNB ++	IFNB +++	TRAIL*	TRAIL**
1	10-15%	80-85%	95-100%	60%	40%	0%	0%	95-100%
2	25-30%	80-85%	95-100%	70%	30%	0%	0%	95-100%
3	10-15%	90-95%	95-100%	90%	10%	0%	5-10%	95-100%
4	10-15%	80-85%	90-95%	90%	10%	0%	0%	95-100%
5	15-20%	85-90%	95-100%	80%	20%	0%	0%	95-100%
6	25-30%	85-90%	90-95%	60%	40%	0%	0%	80-85%
7	40-45%	85-90%	85-90%	60%	40%	0%	0%	70-75%
8	45-50%	80-80%	90-95%	70%	30%	0%	0%	75-80%
9	45-50%	90-95%	90-95%	70%	30%	0%	10-15%	80-85%
10	55-60%	55-60%	65-70%	50%	40%	10%	25-30%	65-70%
11	45-50%	60-65%	70-755	60%	30%	10%	15-20%	75-80%
12	50-55%	75-80%	70-75%	60%	40%	0%	0%	95-100%
13	60-65%	40-45%	60-65%	50%	35%	15%	35-40%	55-60%
14	70-75%	35-40%	35-40%	20%	30%	50%	65-70%	25-30%
15	80-85%	35-40%	35-40%	30%	50%	20%	85-90%	15-20%
16	90-95%	40-45%	35-40%	10%	40%	50%	85-90%	10-15%
17	90-95%	25-30%	30-35%	20%	50%	30%	75-80%	15-20%

USP6: cytoplasmic positivity
p27: nuclear positivity
p16: cytoplasmic positivity
IFNB: intensity of cytoplasmic pozitivity
TRAIL*: nuclear positivity
TRAIL**: membranous positivity

Supplementary Table 2. Relationship between USP6-FISH (%) and different gene expressions								
**Data table – GUSB**								
FISH USP6(%)	USP6	TRAIL	IFNB	JAK1	STAT1	STAT3	JUN	CDKN2A
14	3822	5.7	4.9	3.0	8.6	1.7	2.5	5.7
17	30574	34.3	17.1	29.9	84.4	10.6	22.6	4.3
18	2896	3.5	3.2	2.8	7.0	1.9	3.5	5.3
20	65536	5.7	90.5	16.0	45.3	7.5	26.0	3.0
27	11585	11.3	14.9	5.3	24.3	3.7	8.6	13.0
33	4705	5.7	16.0	8.0	22.6	4.0	14.9	0.7
38	10086	8.0	16.0	7.0	21.1	4.9	3.2	4.6
46	5405	13.0	2.5	3.0	12.1	1.9	2.3	3.2
47	194	5.3	2.5	5.7	8.6	3.5	9.8	8.6
52	891	7.0	2.3	4.0	5.3	2.5	1.3	3.7
58	478	9.2	26.0	4.0	36.8	3.7	4.9	7.0
61	2702	6.5	1.7	3.7	11.3	2.8	3.0	4.3
63	2048	6.1	1.3	2.5	7.0	2.3	1.0	1.5
78	6208	9.2	4.0	4.6	22.6	2.3	24.3	7.5
80	446	3.0	39.4	4.3	4.0	1.7	4.6	16.0
84	5793	1.5	16.0	4.9	3.5	2.5	6.5	7.5
91	549	2.5	13.9	4.6	3.7	3.5	4.3	4.0
**Data table – HPRT1**								
FISH USP6(%)	USP6	TRAIL	IFNB	JAK1	STAT1	STAT3	JUN	CDKN2A
14	1911	2.8	2.5	1.5	4.3	0.9	1.2	2.8
17	1552	1.7	0.9	1.5	4.3	0.5	1.1	0.2
18	1098	1.3	1.2	1.1	2.6	0.7	1.3	2.0
20	7132	0.6	9.8	1.7	4.9	0.8	2.8	0.3
27	3327	3.2	4.3	1.5	7.0	1.1	2.5	3.7
33	1351	1.6	4.6	2.3	6.5	1.1	4.3	0.2
38	1448	1.1	2.3	1.0	3.0	0.7	0.5	0.7
46	1783	4.3	0.8	1.0	4.0	0.6	0.8	1.1
47	52	1.4	0.7	1.5	2.3	0.9	2.6	2.3
52	256	2.0	0.7	1.1	1.5	0.7	0.4	1.1
58	84	1.6	4.6	0.7	6.5	0.7	0.9	1.2
61	776	1.9	0.5	1.1	3.2	0.8	0.9	1.2
63	955	2.8	0.6	1.1	3.2	1.1	0.5	0.7
78	1098	1.6	0.7	0.8	4.0	0.4	4.3	1.3
80	97	0.7	8.6	0.9	0.9	0.4	1.0	3.5

84	1448	0.4	4.0	1.2	0.9	0.6	1.6	1.9
91	97	0.4	2.5	0.8	0.7	0.6	0.8	0.7
**Normalization of real-time PCR considering GUSB and HPRT1 control genes**								
FISH USP6(%)	USP6	TRAIL	IFNB	JAK1	STAT1	STAT3	JUN	CDKN2A
14	2548	3.8	3.3	2.0	5.7	1.2	1.6	3.8
17	2954	3.3	1.7	2.9	8.2	1.0	2.2	0.4
18	1592	1.9	1.8	1.6	3.8	1.0	1.9	2.9
20	12863	1.1	17.8	3.1	8.9	1.5	5.1	0.6
27	5169	5.0	6.7	2.4	10.8	1.7	3.8	5.8
33	2099	2.5	7.1	3.6	10.1	1.8	6.7	0.3
38	2533	2.0	4.0	1.7	5.3	1.2	0.8	1.2
46	2681	6.4	1.2	1.5	6.0	0.9	1.1	1.6
47	82	2.2	1.0	2.4	3.6	1.5	4.2	3.6
52	398	3.1	1.0	1.8	2.4	1.1	0.6	1.7
58	144	2.8	7.8	1.2	11.0	1.1	1.5	2.1
61	1206	2.9	0.8	1.7	5.0	1.3	1.4	1.9
63	1303	3.9	0.8	1.6	4.4	1.5	0.6	1.0
78	1865	2.8	1.2	1.4	6.8	0.7	7.3	2.2
80	159	1.1	14.1	1.5	1.4	0.6	1.6	5.7
84	2317	0.6	6.4	2.0	1.4	1.0	2.6	3.0
91	165	0.7	4.2	1.4	1.1	1.0	1.3	1.2

Supplementary Table 3 Relationship between USP6-FISH (%) and mitosis, lifetime of NF, size

Original data table

FISH USP6(%)	mitosis	lifetime of NF [month]	size [mm]
14	0.5	> 3	21
17	0.5	> 3	9
18	1	2-3	16
20	1	> 3	10
27	1	> 3	13
33	1	2-3	20
38	2	2-3	5
46	5	2-3	12
47	3	2-3	9
52	3	1-2	8
58	6	1-2	7
61	4	2-3	25
63	3	1-2	18
78	5	< 1	25
80	10	< 1	15
84	11	< 1	17
91	18	< 1	15

Modified data table (for statistics)

FISH USP6(%)	mitosis	lifetime of NF [month]	size [mm]
14	0.5	3.0	21
17	0.5	3.0	9
18	1	2.5	16
20	1	3.0	10
27	1	3.0	13
33	1	2.5	20
38	2	2.5	5
46	5	2.5	12
47	3	2.5	9
52	3	1.5	8
58	6	1.5	7
61	4	2.5	25
63	3	1.5	18
78	5	1.0	25
80	10	1.0	15
84	11	1.0	17
91	18	1.0	15

Supplementary Table 4. Detailed NGS data of the selected three NF cases.

sample_ID	Position	End Position	Cytoband	Reference Allele	Sample Allele	Variation Type	Gene Region	Gene Symbol	Transcript ID	Transcript Variant	Protein Variant	Variant Findings	Case Samples	Inferred Activity	Genotype	Sample Genotype Quality	Compound Heterozygous	Call Quality	Read Depth	Allele Fraction	Sample Upstream Filtering	Translation Impact	Classification	SIFT Function Prediction	SIFT Score	PolyPhen-2 Function Prediction	CADD Score	Conservation phyloP p-value	Regulatory Site	Regulator	Transcriptional Regulator	TFBS Enhancer Binding Score	Enhancer Binding Site	microRNA Regulator	Gene Fusion	Fused to	dbSNP ID	DGV ID	Odds Ratio	Association p-value	AFC Frequency	gnomAD Frequency	HGMD	CLINVAR ID	CentoMD	OMIM Gene	OMIM Phenotype	PGMD	COSMIC ID	Cosmic Frequency
Case_No_1	78444635	78444635	p31.1	A	G	SNV	Promoter; Exonic; ncRNA	**FUBP1; DNAJB4**	ENST0000	c.-803A>G; c.-1403A>G; c.54	p.G18G	36	03025-20	normal	Het	100	No	100	629	58,66	Pass	synonymous	Likely Benign				10.830	3,38E-06	Promoter	POLR2A; G	POLR2A; GTF2F1; ZNF354C						61729829				-	0,864		RCV000958459.1						
Case_No_1	61149554	61149554	p16.1	A	G	SNV	Exonic	**REL**	ENST0000	c.1648A>G; c.1744A>G	p.N550D; p.N582D		03025-20	normal	Het	100	No	100	583	44,08	Pass	missense	Uncertain Significance	Tolerated	0,79	Benign	12.140										1,322E+09				-	0								
Case_No_1	212587119	212587119	q34	T	C	SNV	Exonic	**ERBB4**	ENST0000	c.882A>G	p.P294P	7	03025-20	loss	Het	100	No	100	285	44,91	Pass	synonymous	Uncertain Significance				14.410	1,61E-10	Splice Site Loss								77309171				-	0,421		RCV000881984.2						
Case_No_1	215593522	215593522	q35	T	C	SNV	Exonic; ncRNA; 3'UTR	**BARD1**	ENST0000	c.859A>G; c.*878A>G; c.673	p.I738V; p.I719V; p.I287V; p.I268V; p.I225V;	37	03025-20	normal	Het	100	No	100	676	47,34	Pass	missense	Likely Benign	Tolerated	0,53	Benign	18.470										61754118				-	0,747		RCV00075	328920					
Case_No_1	215632255	215632256	q35	CA	TG	Substitution	Exonic; Intronic; ncRNA; 3'UT	**BARD1**	ENST0000	n.1426_1427delTGinsCA; c.	p.V488M; p.V507M; p.V371M	26	03025-20	gain	Het	100	No	100	492	45,53	Pass	missense	Benign	Damaging	0,04	Benign	10.760										386654966				-				243097					
Case_No_1	136062743	136062743	q22.3	C	A	SNV	Exonic	**STAG1**	NM_0058	c.3377G>T	p.S1126I		03025-20	normal	Het	100	No	100	370	40,27	Pass	missense	Uncertain Significance	Tolerated	0,11	Benign	23.600	1,9E-05									764146648				-	0,001								
Case_No_1	149495391	149495396	q32	GCTCTG		Deletion	Exonic	**PDGFRB**	ENST0000	c.2768_2773delCAGAGC; c.:	p.P1084_E1085del; p.P1020_E1021del; p.P923_E924de		03025-20	loss	Het	100	No	100	1081	1,48	Pass	in-frame	Uncertain Significance				11.780										779686590				-									
Case_No_1	26197109	26197109	p22.2	C	T	SNV	Exonic; 3'UTR	**H3C4; AL031777.2**	ENST0000	c.*261G>A; c.370G>A	p.D124N	75	03025-20	normal	Het	100	No	100	959	46,19	Pass	missense	Uncertain Significance	Damaging	0,01		23.900		ENCODE T	POLR2A	POLR2A						367717762				-	0,004								
Case_No_1	30668352	30668352	p21.33	G	A	SNV	Exonic	**MDC1**	NM_01464	c.6160C>T	p.R2054W	3	03025-20	normal	Het	100	No	100	589	48,9	Pass	missense	Uncertain Significance	Damaging	0	Probably Damagi	22.800										538887974				-	0,002							6951199	0,08
Case_No_1	32181017	32181017	p21.32	T	C	SNV	ncRNA; Exonic	**NOTCH4**	NR_13494	c.2333A>G; n.2472A>G; n.2!	p.N778S		03025-20	normal	Het	100	No	100	627	47,53	Pass	missense	Uncertain Significance	Damaging	0	Benign	23.200	8,22E-05									377703621				-	0,002								
Case_No_1	33287796	33287796	p21.32	G	C	SNV	Exonic	**DAXX**	ENST0000	c.1493C>G; c.1457C>G; c.12	p.A498G; p.A411G; p.A486G	3	03025-20	normal	Het	100	No	100	1243	48,19	Pass	missense	Likely Benign	Tolerated	0,26	Benign	< 10										146304558				-	0,833		RCV000888329.1						
Case_No_1	151857451	151857451	q25.1	A	]6:152023138]	Fusion	Splice Site	**CCDC170**	NM_0250	ESR1 - CCDC170:G58_A2148	ESR1 - CCDC170:E20_*716	1	03025-20	normal	No-Call	-	No		6		Pass		Uncertain Significance					1,52E-05	Splice Site Loss						**ESR1-CCDC170**	**ESR1**														
Case_No_1	32406284	32406286	p12	AAG		Deletion	Exonic; Intronic	**NRG1**	ENST0000	c.746-47056_746-47054delA	p.K16del		03025-20	normal	Het	100	No	100	761	41,66	Pass	in-frame	Uncertain Significance																		-									
Case_No_1	32406290	32406290	p12	A	G	SNV	Exonic; Intronic	**NRG1**	ENST0000	c.46A>G; c.38-47056A>G; c.	p.K16E		03025-20	normal	Het	100	No	100	734	1,91	Pass	missense	Uncertain Significance	Tolerated	0,08	Benign	23.700	0,002704																						
Case_No_1	41906160	41906160	p11.21	C	G	SNV	Exonic	**KAT6A**	ENST0000	c.336G>C	p.E112D	1	03025-20	normal	Het	100	No	100	853	49,82	Pass	missense	Uncertain Significance	Tolerated	0,17	Benign	21.400	2,59E-08									138399992				-	0,12								
Case_No_1	48691068	48691068	q11.21	G	A	SNV	Exonic	**PRKDC**	NM_0010	c.11594T>C *; c.11805C>T; c	p.L3896P; p.G3935G; p.L3865P; p.G3904G	6	03025-20	loss	Het	100	No	100	637	46,31	Pass	missense; synonymous	Likely Benign				< 10	4,06E-08									8178249				-	0,557		RCV000528197.4; RCV000434056.2					9161282	0,71
Case_No_1	20621982	20621983	p21.3		G	Insertion	Promoter; Intronic	**MLLT3**	ENST0000	c.12+261dupC; c.-209dupC		5	03025-20	normal	Het	100	No	100	433	43,88	Pass		Uncertain Significance				16.940		Promoter	ETS1; POL	ETS1; POLR2A; MYC						1,463E+09				-	0								
Case_No_1	108098576	108098576	q22.3	C	G	SNV	Exonic	**ATM**	NM_00135	c.146C>G	p.S49C	246	03025-20	loss	Het	100	No	100	589	49,41	Pass	missense	Benign	Damaging	0	Possibly Damagin	23.600										1800054				-	0,718	CM990201	RCV00012	188310	607585	114480		6495406	4,49
Case_No_1	50480102	50480102	q13.12	G	T	SNV	Exonic	**SMARCD1**	ENST0000	c.336G>T	p.Q112H	4	03025-20	normal	Het	100	No	100	678	46,02	Pass	missense	Uncertain Significance	Damaging	0,03	Benign	19.830	4,46E-08									139120093				-	0,281								
Case_No_1	57858964	57858964	q13.3	T	C	SNV	Exonic	**GLI1**	NM_0011	c.460T>C; c.337T>C; c.76T>C	p.C113R; p.C26R; p.C154R		03025-20	normal	Het	100	No	100	587	50,6	Pass	missense	Uncertain Significance	Tolerated	0,07	Possibly Damagin	22.800	0,000252									202066753				-	0,008								
Case_No_1	72831629	72831629	q22.2	G	A	SNV	Exonic	**ZFHX3**	ENST0000	c.4952C>T; c.2210C>T	p.T737M; p.T1651M	21	03025-20	normal	Het	100	No	100	857	51,93	Pass	missense	Uncertain Significance	Tolerated	0,09	Benign	22.100	0,001648									117283459				-	0,086		RCV000996322.1						
Case_No_1	30264461	30264461	q11.2	G	A	SNV	Exonic	**SUZ12**	ENST0000	c.196G>A	p.A66T	4	03025-20	normal	Het	100	No	100	904	46,68	Pass	missense	Uncertain Significance			Benign	21.700	3,8E-06									199728576				-	0,05							3180169	1,79
Case_No_1	37687481	37687481	q12	C	G	SNV	Exonic	**CDK12**	ENST0000	c.4358C>G; c.4385C>G	p.P1462R; p.P1453R		03025-20	normal	Het	100	No	100	885	47,01	Pass	missense	Uncertain Significance	Damaging	0,01	Benign	23.100																							
Case_No_1	45856552	45856552	q13.32	A	C	SNV	Exonic	**ERCC2**	ENST0000	c.1706T>G; c.1472T>G	p.I569S; p.I491S		03025-20	loss	Het	100	No	100	702	49,29	Pass	missense	Uncertain Significance	Damaging	0	Probably Damagi	31.000	6E-08																						
Case_No_1	45912731	45912732	q13.32		GAA	Insertion	Exonic; 3'UTR	**ERCC1; CD3EAP**	ENST0000	c.1513_1515dupAAG; c.*19	p.K505dup; p.K503dup		03025-20	loss	Het	100	No	100	619	5,49	Pass	in-frame	Uncertain Significance				9.729	0,000306									759287808				-	0,002								
Case_No_1	31024698	31024698	q11.21	C	G	SNV	Exonic; Intronic	**ASXL1**	ENST0000	c.4000C>G; c.4183C>G; c.41	p.L1334V; p.L1390V; p.L1395V	17	03025-20	normal	Het	100	No	100	841	49,46	Pass	missense	Uncertain Significance	Tolerated	0,18	Probably Damagi	16.940	0,003236									150004862				-	0,044		RCV000896760.2					41990	0,49
Case_No_1	41514552	41514552	q12	G	C	SNV	Exonic	**PTPRT**	NM_0070	c.109C>G	p.H37D		03025-20	normal	Het	100	No	100	460	46,3	Pass	missense	Uncertain Significance	Tolerated	0,88	Benign	18.660										200648521				-	0,044								
Case_No_1	29682903	29682903	q12.2	C	G	SNV	Intronic	**EWSR1**	ENST0000	c.414-9C>G; c.582-9C>G; c.600-9C>G		2	03025-20	normal	Het	100	No	100	381	42,78	Pass		Uncertain Significance				< 10		Splice Site Loss								189387468				-	0,281		RCV000953405.2; RCV001199247.1						
Case_No_14	120484321	120484321	p12	A	T	SNV	Exonic	**NOTCH2**	NM_0012	c.2809T>A	p.C937S		40112-18	loss	Het	91	No	91	172	4,07	Pass	missense	Uncertain Significance	Damaging	0	Probably Damagi	29.900																							
Case_No_14	156830779	156830779	q23.1	G	A	SNV	Promoter; Exonic; Intronic	**NTRK1; INSRR**	ENST0000	c.53G>A; c.-2366C>T; c.123-	p.G18E	29	40112-18	normal	Het	100	No	100	86	52,33	Pass	missense	Benign	Tolerated	0,08	Benign	19.220		ENCODE T	CCNT2; ET	CCNT2; ETS1; E2F6						1007211				-	0,799	CM201410	RCV000173175.4; RCV000757575.5; RCV001081416.2						
Case_No_14	158614141	158614141	q23.1	G	A	SNV	Exonic	**SPTA1**	NM_00312	c.4240C>T	p.R1414C	5	40112-18	normal	Het	100	No	100	151	45,7	Pass	missense	Uncertain Significance	Damaging	0	Probably Damagi	29.100										201399968				-	0,027	CM192011	RCV001097422.1; RCV001097421.1; RCV001097423.1						
Case_No_14	215632255	215632256	q35	CA	TG	Substitution	Exonic; Intronic; ncRNA; 3'UT	**BARD1**	ENST0000	n.1426_1427delTGinsCA; c.	p.V488M; p.V507M; p.V371M	26	40112-18	gain	Het	100	No	100	118	99,15	Pass	missense	Benign	Damaging	0,04	Benign	10.760										386654966				-				243097					
Case_No_14	32188010	32188010	p21.32	T	A	SNV	ncRNA; Exonic	**NOTCH4**	NR_13494	n.1350A>T; c.1211A>T	p.D404V		40112-18	normal	Het	100	No	100	181	49,17	Pass	missense	Uncertain Significance	Damaging	0,01	Possibly Damagin	22.700	0,000378									144043459				-	0,211								
Case_No_14	101921292	101921292	q22.1	A	C	SNV	Exonic	**CUX1**	ENST0000	c.1630A>C; c.1498A>C; c.15	p.K507Q; p.K546Q; p.K500Q; p.K544Q; p.K5	16	40112-18	normal	Het	100	No	100	195	47,18	Pass	missense	Uncertain Significance	Tolerated	0,07	Probably Damagi	26.100	2,13E-09									118010189				-	0,548								
Case_No_14	108181014	108181014	q22.3	A	G	SNV	Exonic; Intronic	**C11orf65; ATM**	NM_00135	c.641-1216T>C; c.*39-1216T	p.K1964E	41	40112-18	loss	Het	100	No	100	80	45	Pass	missense	Likely Pathogenic	Tolerated	0,22	Benign	22.200	0,000499									201963507				-	0,009	CM091051	RCV000585940.3; RCV000197688.9; RCV000212034.4;					2110551	4.76; 1.81
Case_No_14	50480102	50480102	q13.12	G	T	SNV	Exonic	**SMARCD1**	ENST0000	c.336G>T	p.Q112H	4	40112-18	normal	Het	100	No	100	183	43,72	Pass	missense	Uncertain Significance	Damaging	0,03	Benign	19.830	4,46E-08									139120093				-	0,281								
Case_No_14	2213943	2213943	p13.3	C	T	SNV	Exonic	**TRAF7**	ENST0000	c.22C>T	p.R8C	1	40112-18	normal	Het	100	No	100	181	48,07	Pass	missense	Uncertain Significance			Probably Damagi	23.900	0,003373									772309846				-	0,002							6986165	2.94
Case_No_14	40476837	40476837	q21.2	T	C	SNV	Exonic	**STAT3**	NM_00138	c.1492A>G; c.1198A>G	p.I498V; p.I400V	3	40112-18	normal	Het	100	No	100	206	41,75	Pass	missense	Uncertain Significance	Tolerated	0,31	Benign	16.860										146620441				-	0,027		RCV000653285.3						
Case_No_14	30301598	30301598	q12	A		Deletion	Promoter	**CCNE1**	NM_00123	c.-1485delA; c.-1483delA; c.-2019delA			40112-18	normal	Het	100	No	100	280	3,21	Pass		Uncertain Significance						Promoter	Gata1	Gata1																			
Case_No_14	30301602	30301602	q12	A	G	SNV	Promoter	**CCNE1**	NM_00123	c.-1482A>G; c.-1480A>G; c.-2016A>G			40112-18	normal	Het	90	No	90	291	2,75	Pass		Uncertain Significance				< 10		Promoter	Gata1; HN	Gata1; HNF1B						111343903													
Case_No_15	113991999	113991999	q13	G	A	SNV	Promoter; Intronic	**PAX8; PAX8-AS1**	ENST0000	n.-1105G>A; n.-1847G>A; c.1008+972C>T; c.1087+972C>T; c.777+7129C>T			40480-18	normal	Het	100	No	100	49	48,98	Pass		Uncertain Significance				< 10		Promoter	Hltf; Esrrb	Hltf; Esrrb; NFIC						181163509				-	0,166								
Case_No_15	141458182	141458182	q22.1	C	A	SNV	Exonic	**LRP1B**	NM_01855	c.6436G>T	p.V2146F	6	40480-18	normal	Het	100	No	100	14	57,14	Pass	missense	Likely Benign	Damaging	0,01	Benign	22.000	0,001995									145744070				-	0,342		RCV000891192.1						
Case_No_15	215632255	215632256	q35	CA	TG	Substitution	Exonic; Intronic; ncRNA; 3'UT	**BARD1**	ENST0000	n.1426_1427delTGinsCA; c.	p.V488M; p.V507M; p.V371M	26	40480-18	gain	Hom	0	No	100	35	100	Pass	missense	Benign	Damaging	0,04	Benign	10.760										386654966				-				243097					
Case_No_15	1806087	1806087	p16.3	C	T	SNV	Exonic; Intronic; ncRNA	**FGFR3**	NM_00014	n.1532C>T; c.1106C>T; c.11	p.A371V; p.A369V	1	40480-18	normal	Het	100	No	100	90	51,11	Pass	missense	Uncertain Significance	Tolerated	0,58	Benign	13.440										146970233				-	0,003		RCV001215293.1						
Case_No_15	153247289	153247289	q31.3	G	A	SNV	Exonic	**FBXW7**	ENST0000	c.1513C>T; c.1159C>T; c.127	p.R387C; p.R425C; p.R505C	389	40480-18	loss	Het	100	No	100	75	4	Pass	missense	Pathogenic	Damaging	0,01	Probably Damagi	26.700										149680468				-	0		RCV000444218.1; RCV000433395.1; RCV000437170.1;					33844; 229	1.14; 1.24
Case_No_15	149471124	149472098	q32		DEL:975	Large Deletion	Intronic; 5'UTR; Splice Site in	**CSF1R**	ENST00000286301.7; NR_164679.1; NM_001375321.1; NM_001349736.1; NM_001375320.1; NI				40480-18	loss	Het-Amb	-	No	1	4		Pass		Uncertain Significance															nsv830518; esv22778												
Case_No_15	176512319	176512319	q35.2	G	A	SNV	Promoter	**FGFR4**	NM_21364	c.-1769G>A; c.-1813G>A; c.-1754G>A			40480-18	normal	Het	100	No	100	50	50	Pass		Uncertain Significance				< 10		Promoter	En1; MZF1	En1; MZF1_1-4						142097461				-	0,437								
Case_No_15	32163409	32163409	p21.32	C	G	SNV	Promoter; Exonic; ncRNA	**GPSM3; NOTCH4**	ENST0000	n.5423G>C; n.5525G>C; c.58	p.G1939G	99	40480-18	normal	Het	100	No	100	58	48,28	Pass	synonymous	Likely Benign				< 10		Promoter	CTCF; SMC	CTCF; SMC3; ETS1; TBP; EBF1; FOS; RFX5; NRF1; GATA1; POLR2A; Mafb; RAD21; ZBTB7A; TFAP2A; NFYA; NFYB; POU2F2; CTCFL; SP2; SP1; TAF1																				
Case_No_15	41903782	41903783	p21.1	AG	CA	Substitution	Exonic	**CCND3**	NM_00113	c.531_532delCTinsTG; c.624	p.S63A; p.S259A; p.S178A; p.S187A; p.S209	7	40480-18	normal	Het	100	No	100	29	55,17	Pass	missense	Uncertain Significance	Tolerated	0,87	Benign	9.550										386700585				-							GV000002955		
Case_No_15	79082361	79082361	q21.11	G	C	SNV	Promoter; Exonic; ncRNA	**MAGI2-AS3; MAGI2**	NR_03834	n.-915G>C; c.276C>G; n.89G	p.P92P	2	40480-18	normal	Het	100	No	100	59	35,59	Pass	synonymous	Likely Benign				16.820	2,2E-10	Promoter	TFAP2A; N	TFAP2A; MZF1_5-13; ELK1; ETS1						146012909				-	0,659		RCV00008	235742					
Case_No_15	92404051	92404051	q21.2	C	T	SNV	Exonic	**CDK6**	NM_00125	c.328G>A	p.D110N	7	40480-18	gain	Het	100	No	100	26	65,38	Pass	missense	Uncertain Significance	Damaging	0,05	Benign	23.000	1,99E-08									35654944				-	0,146		RCV000500251.1; RCV000971369.2						
Case_No_15	48701786	48701786	q11.21	A	T	SNV	Exonic	**PRKDC**	NM_00108	c.10684T>A; c.10566G>T *	p.L3522L; p.L3562M	4	40480-18	loss	Het	100	No	100	18	50	Pass	missense; synonymous	Benign	Damaging	0	Probably Damagi	24.100										8178232				-	0,695		RCV000557472.4; RCV000506229.1						
Case_No_15	8486299	8486299	p24.1	G	A	SNV	Exonic; Intronic	**PTPRD**	NM_13035	c.1814-972C>T; c.1805-975C	p.L840F		40480-18	normal	Het	100	No	100	32	50	Pass	missense	Uncertain Significance	Tolerated	0,08	Probably Damagi	29.400										142009246				-	0,018								
Case_No_15	133252760	133252760	q24.33	A	C	SNV	Exonic	**POLE**	ENST0000	c.940T>G; c.859T>G	p.S314A; p.S287A	14	40480-18	loss	Het	100	No	100	50	48	Pass	missense	Likely Pathogenic	Tolerated	0,12	Benign	22.000										770403791				-	0,01	CM192268	RCV000474943.6; RCV000709278.1; RCV000657078.4; RCV000483379.3; RCV000210168.1						
Case_No_15	3819294	3819294	p13.3	C	T	SNV	Exonic	**CREBBP**	NM_00438	c.2827G>A; c.2941G>A	p.A943T; p.A981T	25	40480-18	normal	Het	100	No	100	59	45,76	Pass	missense	Likely Benign	Tolerated	0,77	Benign	16.460										61753380				-	0,34	CM021081	RCV000715867.1; RCV000509267.1; RCV000870748.2;					9213242	9.09
Case_No_15	89348973	89348973	q24.3	G	A	SNV	Exonic	**ANKRD11**	NM_00125	c.3977C>T	p.T1326M	4	40480-18	normal	Het	100	No	100	52	40,38	Pass	missense	Uncertain Significance	Damaging	0,04	Benign	17.630										144673419				-	0,059		RCV000436122.3; RCV001078483.1; RCV000719739.1						
Case_No_15	56437548	56437548	q22	T	C	SNV	Exonic	**RNF43**	ENST0000	c.533A>G; c.914A>G; c.791A	p.Q264R; p.Q178R; p.Q305R	2	40480-18	normal	Het	100	No	100	41	46,34	Pass	missense	Uncertain Significance	Damaging	0,04	Probably Damagi	25.900	3,19E-08									889865921				-	0							4755833	1.67
